# Attitudes and Health Behavior of Lawyers in Coimbatore, Tamil Nadu

**DOI:** 10.1155/2015/616719

**Published:** 2015-07-15

**Authors:** G. Barani, Pavithra Sabapathy

**Affiliations:** Department of Management Studies, Anna University Regional Centre Coimbatore, Coimbatore, Tamil Nadu 641 046, India

## Abstract

The aim of the study is to evaluate the differences in the behavior and attitudes of male and female lawyers regarding their lifestyles and health habits. Lawyers were randomly chosen. Data was obtained through a structured questionnaire distributed among the lawyers of Coimbatore district, Tamil Nadu. Lawyers are found to have unfavorable health practices related to use of tobacco and alcohol, exercise, diet, sleeping habits, and stress. This resulted in obesity, depression, and blood pressure. Many lawyers reported use of alcohol regularly, even as often as every day, and nearly half of them smoked. Many of the lawyers had poor feeding habit of skipping meals and eating snacks as breakfast. Most lawyers considered stressful situations to be unavoidable. Thus identifying individual lawyers with poor health behaviors and providing medical help are essential.

## 1. Introduction

A lawyer's day to day activities and attitudes have a major effect on their health. Physical activity, diet, and psychosocial aspects like hygiene, stress, smoking, and alcohol usage play a vital role in sustaining healthiness and preventing illness. The incidence of substance abuse (both alcohol and drugs) is higher for lawyers than the population as a whole [1]. The varying lifestyle and food habits of male and female lawyers along with a decline in physical activity lead to sickness. These conditions automatically decline the quality of life. The practice of law can be demanding and exceedingly stressful. It is a well-known fact that an inactive lifestyle connected with a little physical activity has become a severe health concern. Physical activity can extensively reduce the threat of many problems like obesity, diabetes, and stress related issues. Three times more law students than medical students were found smoking. Regarding alcohol usage, no significant differences were found between medical and law student's behavior [2].

The lawyers are found to undergo stress more than other professionals as they deal with the problems of others. Such stressors, in combination with changing lifestyles, support the development of risky lifestyles, for example, high consumption of alcohol and drugs, and a low concern for healthy nutrition and restful sleep. Although some of these practices may be transient in nature, other “habits” could persevere into middle and old age to cause health hazards later in life. Likelihood of depression is three times higher for lawyers than other employed people, and the reasons include the pressures of the job and characteristics that make lawyers good at their jobs. Lawyers are found to suffer from the symptoms of depression, anxiety, obsessive-compulsive disorders, and other problems. The author adds that, of every five lawyers, who required help, two have psychological issues, one suffers from addiction, and two have both problems [3]. Flexible work arrangements are one way firms can reduce stress levels among their lawyers, promote work-life balance, and, ultimately, avoid productivity costs associated with unaddressed mental illness in the workplace [4].

This study enquired the details pertaining to respondent's demographic characteristics, food consumption patterns, sleeping habits, and smoking, drinking and drugs, and stress.

According to the World Health Organization [5], physical activity is defined as any bodily movement produced by skeletal muscles that require energy expenditure. Physical inactivity (lack of physical activity) has been identified as the fourth leading risk factor for global mortality.

Eating healthy, balanced food on a regular basis helps in maintaining a balanced weight, good mood, enlarged energy levels, positive stimulation to others, and prospective for heightening the quality of life. Food consumption patterns vary significantly, as females are found to be more conscious than males [6]. Poor sleep has been linked to many medical conditions, including obesity. Improper sleeping habits also result in tiredness and lack of concentration in work. Good sleeping is associated with good health [7].

Studies have proved that smoking decreases life span by seven to eight years.It is been found thatsmoking rate of lawyers was found to be higher than that of medical doctors [8]. Consumption of alcohol may cause stroke, high blood pressure, liver diseases, stroke, some types of cancer, and so forth. As lawyers undergo a high level of stress, majority of lawyers are found to consume alcohol. Alcohol usage and stress are interlinked; as the amount of stress increases, the usage of alcohol also increases [9]. Source of stress for some lawyers is the adversarial nature of the profession itself [10]. Lawyers are typically involved in business disputes or deals when the stakes are high, and the downside is great, so there is more reason for stress. Women lawyers are found to undergo more stress than male lawyers [11].

The findings of the study indicate that male lawyers have positive health behaviors in regard of physical activity and stress. Male lawyers are found to have a considerable physical activity and undergo less stress than female lawyers, whereas female lawyers are found to have constructive health behavior in terms of eating habits and usage of tobacco and drugs. Female lawyers are found to have good eating habits and only few women lawyers are found to have drug and smoking habits. But both male and female lawyers are found to have negative impact in terms of sleeping habits. Thus, both male and female lawyers do not get adequate sleep.

Hence the objectives of the study are to

- assess the prevalence of a range of health behavior variables and lifestyle characteristics (e.g., food consumption patterns; physical activity; restful sleep; tobacco smoking; alcohol consumption; stress) of the lawyers by gender,
- compare the health behaviors and lifestyle characteristics of male and female lawyers.

Thus, the main aim of the study is to find out the discrepancy that prevails between male and female lawyers in terms of their behavior and attitude towards their lifestyle and health habits.

## 2. Materials and Methods

### 2.1. Participants

The study was conducted among a group of 100 lawyers in Coimbatore district. The respondents were selected using convenience sampling method. The study units that happen to be available at the time of data collection are selected in the sample. Thus, those who were reachable and approachable were selected for the study. A descriptive research was carried out in Coimbatore. Interview schedule was used to collect the data regarding health behavior of lawyers.

### 2.2. Questionnaire

The anonymous questionnaire consisted of 30 questions (including 25 closed-ended and 5 open-ended questions). Open-ended questions were used to make it easy for the respondents to freely express their response to the given questions. The first part of the questionnaire contained personal information like age, gender, marital status and children, living arrangements, financial sufficiency, and educational details. It was followed by questions that dealt with issues such as physical activity, physical fitness, nutrition, personal hygiene, alcohol and tobacco use, stress, and gaining knowledge on health.

An information sheet accompanied each questionnaire outlining the research aims and objectives of the study. All data were confidential and data protection was observed at all stages of the study. The obtained results were characterized by descriptive statistics.

### 2.3. Statistical Methods

The statistical analysis was done using SPSS. The data that was collected was analyzed using percentage analysis and Chi-Square test.

## 3. Results

Table 1 describes the percentage analyses of the various factors and their constructs used in this study.

Table 1 shows the cumulative percentage analysis of the lawyers' opinions towards the factors affecting health. On examining Table 1, for the first variable food consumption patterns, majority of the respondents have opted for “agree” option, from which it can be inferred that most of the respondents agree that food consumption patterns have a direct effect on health. For the second variable physical activity, most of the respondents have chosen “agree” category, from which it can be inferred that majority of the respondents agree that physical activity plays an important role in maintaining good health. For the third variable sleeping habits, most of the respondents' opinions were “strongly agree,” from which it can be assumed that majority of the respondents strongly agree that improper sleeping habits affect health. For the fourth variable usage of tobacco, majority of the respondents have opted for “strongly agree” category, from which it can be concluded that majority of the respondents strongly agree that usage of tobacco affects health. For the fifth variable usage of drugs, “strongly agree” was major respondent's option, which indicates that most of the respondents feel that using drugs has a negative effect on health. With regard to stress, which was the last variable, most of the respondents' choices were “agree,” which shows majority of the respondents agree that stress has an impact on health. Thus, from Table 1, it can be concluded that lawyers were found to be aware of effects of health due to improper behaviors like worst food consumption patterns, less physical activity, improper sleeping habits, usage of drugs and tobacco, and increased stress.

Table 2 shows the aggregate percentage of respondents' opinions towards their perception towards self-responsibility for health. On examining Table 2, most of the responses fall under agree and neither agree nor disagree categories with the factors for the first category, “There is much I can do for myself to promote my own health.” For the second variable “If am going to get sick, I will get sick no matter what I do,” most of the respondents' responses were “strongly agree,” while the same number of people responded with disagree and neither agree nor disagree category next to it. For the third variable “With all the new drugs and medicines being developed now, people will be able to stay healthier,” most of the responses fall under neither agree nor disagree and agree categories. For the fourth variable “Each adult person is responsible for himself or herself to avoid getting sick,” most of the responses fall under strongly agree and agree categories. For the fifth variable “Though I maintain good health habits, I'll get sick,” most of the responses fall under strongly disagree and disagree groups. For the last variable “If a person gets sick, it is generally not his or her own fault,” majority of the respondents have opted for strongly disagree and disagree categories.

A Chi-Square test was conducted on the dependent and independent variables to identify the statistically significant constructs (if any). The Null and Alternate hypotheses were framed and results were drawn with 0.05 significance value.

Chi-Square test is used to check on the relationship between the below two variables:

- relationship between gender and
  - good consumption patterns,
  - physical activity,
  - sleeping habits,
  - usage of tobacco,
  - usage of drugs,
  - stress.

*Null Hypothesis Ho*. There is no relationship between gender and food consumption patterns, physical activity, sleeping habits, and usage of tobacco and drugs and stress.

Table 3 shows that there is relationship between gender and food consumption patterns, physical activity, sleeping habits, and usage of tobacco and drugs and stress. This reveals that attitudes of men and women differ in their health behavior. Regarding food consumption patterns, women lawyers are found to have good food habits than men lawyers. The importance of eating healthily was rated very important/important by slightly more proportions of female lawyers, while male lawyers were more likely to view eating healthily as not at all important/not important.

## 4. Discussion

The study clearly indicates that physical activity is not a priority among the women lawyers. Male lawyers were generally more likely to have had undertaken exercises tostrengthen or tone their muscles. The unwillingness of women lawyers to take part in various forms of physical activity has effects on their health. Results of the study are supported by the findings of Schuit [12], where physical activity has good health benefits.

It is a well-known fact that a proper diet is a crucial element of good health but does not seem to be regarded as such by male lawyers. Although it is commonly agreed by male lawyers, they do not pay much attention to proper nutrition and are characterized by rather negative eating habits. The nutritional risks specific to lawyers are alarming. It has been found that only a low proportion of lawyers consumed healthy items like fruits and vegetables daily. This result is resistant with the results of the study conducted by Bonauto et al. [13], where they found that those who had good dietary habits are less obese.

As for the amount of sleep, there were no gender differences among lawyers. Both men and women lawyers were found to have less sleep, that is, less than eight hours of sleep per day. Thus, there is no significant relationship between the sleeping habits of men and women lawyers. Findings of the study are supported by Barristers Educational Services [14], where it is found that lawyers are the second most sleep-deprived occupation in the United States.

Information obtained from our research regarding lawyers drinking habits is very worrying. Although we cannot expect lawyers to completely abstain from drinking alcohol the fact that a significant percentage of them drink habitually and even every day must be brought to attention. Men lawyers were found to drink more times in a week than female lawyers whereas only a very few women lawyers were found to drink alcohol occasionally. Thus, there is significant relationship between usage of drugs and gender, as male lawyers are found to consume more alcohol than female lawyers. This result is supported with the findings of Wyshak and Lawrence [15], where alcohol usage is found to have negative impact on lawyers and judges.

Regarding usage of tobacco, higher proportions of male lawyers consumed tobacco daily, and none of the female lawyers reported themselves as smokers. Majority of them are found to smoke more than five times a day. Some of the male lawyers were found to have attempted to quit smoking. This shows there is relationship between gender and tobacco usage, as male lawyers are found to smoke more than women lawyers. This result is supported by Sun et al. [16], where a bigger number of men than women smoke.

Regarding the amount of stress experienced by male and female lawyers, women lawyers are found to experience more stress than male lawyers. As women take care of their household activities along with their professional work, they undergo work-life imbalance and are found to experience more stress. Women lawyers were highly affected by stress more than male lawyers. This view is supported by Leskinen et al. [17], where women lawyers are found to have lesser job satisfaction and high level of stress.

## 5. Conclusion

Overall this research concludes that female lawyers are highly affected by stress and they are found to have positive health practices compared with men. On the other hand, male lawyers were found good at physical activity. But large proportion of the male lawyers engaged in risk taking behaviors such as drinking and smoking, where women lawyers were not.

The studies should be continued in further years to see if any changes in the habits and attitudes of lawyers have taken place, determine the direction of these changes, and develop a strategy that would facilitate improvement in the years to come.

## Figures and Tables

**Table 1:** Percentage analysis of factors affecting health.

Serial number	Factors	Number	SD	D	NA/ND	A	SA
I	Food consumption patterns	100	5	11	23	46	15
II	Physical activity	100	5	4	19	40	32
III	Sleeping habits	100	12	17	21	24	26
IV	Usage of tobacco	100	—	5	11	33	51
V	Usage of drugs	100	—	9	14	33	44
VI	Stress	100	8	14	21	45	12

**Table 2:** Perception towards self-responsibility for health.

Factors	SD	D	NA/ND	A	SA
There is much I can do for myself to promote my own health	—	4	16	48	32
If am going to get sick, I will get sick no matter what I do	12	18	18	16	36
With all the new drugs and medicines being developed now, people will be able to stay healthier	14	11	34	30	11
Each adult person is responsible for himself or herself to avoid getting sick	21	12	15	28	24
Though I maintain good health habits, I'll get sick	34	25	11	12	18
If a person gets sick, it is generally not his or her own fault	32	28	12	16	12

**Table 3:** Chi-Square test.

Serial number	Factors	Calculated value	Degree of freedom	Table value	Significance
1	Food consumption patterns	166.586	4	9.488	Significant
2	Physical activity	237.20	4	9.488	Significant
3	Sleeping habits	0.007	1	3.841	Nonsignificant
4	Usage of tobacco	30.72	1	3.841	Significant
5	Usage of drugs	228.48	1	3.841	Significant
6	Stress	510.168	2	5.991	Significant
